# Balanced Scorecard-Based Hospital Performance Measurement Framework: A Performance Construct Development Approach

**DOI:** 10.7759/cureus.24866

**Published:** 2022-05-09

**Authors:** Ahmad A Abu Jaber, Abdulqadir J Nashwan

**Affiliations:** 1 Emergency Medicine: Nursing, Hamad Medical Corporation, Doha, QAT; 2 Oncology: Hematology, Hamad Medical Corporation, Doha, QAT

**Keywords:** healthcare policy, scale development, performance measurement, organizational performance, balanced scorecard

## Abstract

Introduction

Despite the critical importance of hospital performance measurement, empirically validated hospital performance frameworks lack. The balanced scorecard is considered one of the most influential contributions in the performance measurement literature. Since the introduction of the balanced scorecard in the early 90s, many scholars have used a balanced scorecard to enable hospital performance measurement and improvement. Therefore, this study aimed to construct and validate a balanced scorecard-based hospital performance framework. Additional to the original four perspectives, the quality of care is added as a perspective for the balanced hospital scorecard. It reflects one of the key strategic objectives in any healthcare organization.

Methods

The study adopted a two-phase model to validate the framework empirically. The first is the exploratory phase, where feedback from academicians and professionals helped finalize the framework in the form of scale. In the second phase, the scale was tested for dimensionality, reliability, and validity.

Results

A total of 200 (81 responded, RR= 40%) senior managers working in Hamad Medical Corporation (HMC), the largest healthcare provider in Qatar, were surveyed. The content, convergent, and discriminant validities were established. The study conducted composite reliability and Cronbach's alpha tests for the reliability, and all variables were found to have alpha and composite reliability higher than 0.7.

Conclusion

The findings suggest that senior managers in HMC make a meaningful distinction between the five attributes of hospital performance. Findings, contributions, limitations, directions for future research, and managerial implications are all discussed.

## Introduction

Over time, managers realize that no single measure can provide a clear performance target or focus attention on the critical areas of the business. Managers want a balanced presentation of both financial and operational measures [1, 2]. Organizational performance measurement is of utmost importance for organizations as it determines their ability to manage efficiency, effectiveness, customer value, and competitiveness. An organization's performance is a "fundamental construct in strategic management", according to researchers at the University of British Columbia (UBC) [3].

In 2000, Stewart et al. [4] argued that most of the literature that tackles performance management targeted manufacturing businesses. Therefore, in the context of the healthcare industry, the lack of relevant frameworks that can inform the leaders and the academicians on how to measure and improve the performance of the hospitals, the different orientations, values, and objectives of the healthcare settings, the significant diversity of the stakeholders and the different expectations of the shareholders make it very challenging to construct a comprehensive performance measurement system that can measure the historical performance and influence and improve the performance in the future. Many scholars have constructed frameworks to measure hospital performance a few years after Stewart and Bestor. One of the most influential philosophies that helped organizations design multidimensional performance measurement systems and achieve their strategic goals is the Balanced Scorecard (BSC), introduced by Kaplan and Norton in 1992. Since the introduction of the BSC, many academicians and practitioners in the healthcare industry have reported that BSC is an appropriate tool to measure and drive the performance of hospitals and healthcare organizations [5-7]. Nonetheless, there is still a lack of empirically validated comprehensive performance measurement frameworks tailored to serve hospitals’ leaders to measure and improve hospital performance and achieve the hospital's strategic objectives.

Balanced Scorecard (BSC)

The BSC is a customized performance measurement system (PMS) that goes beyond conventional accounting and is based on organizational strategy. The BSC aims to link long-term strategic objectives with short-term actions in an organization [6][8].

The BSC typically measures the organizational performance across the following four balanced and integrated perspectives:

*Financial perspective*. It is concerned with how shareholders see the organization. It includes “several profitability and growth measures, such as return on sales, return on investment, operating income, and sales growth” [9].

*Customer perspective*. It is concerned with how well an organization serves its customers’ needs. Moreover, this aspect is related to the organization's reputation and how it differentiates itself from its competitors [9]. It includes measures such as “customer satisfaction, customer retention rate, and market share.” [10] discussed that BSC shows how customer satisfaction leads to the acquisition and retention of customers and that these attributes are a precedent for improved market share, customer profitability, and financial goals.

*Internal business perspective*. It is developed after the organization identifies its financial and customer perspectives. It is concerned with how well the organization performs its key internal operational processes. Based on [11], this perspective captures opportunities to improve critical processes to satisfy the customers and achieve operational excellence.

*Learning and growth perspective*. The changes and improvements that an organization needs to adapt to achieve its vision [9]. According to Voelker et al. [6], this perspective relates to an organization’s intangible assets and to the ability to excel in the future. It has measures related to employees' capabilities, satisfaction, motivation, and empowerment.

Proposed BSC in Healthcare Organizations

**Figure 1:**
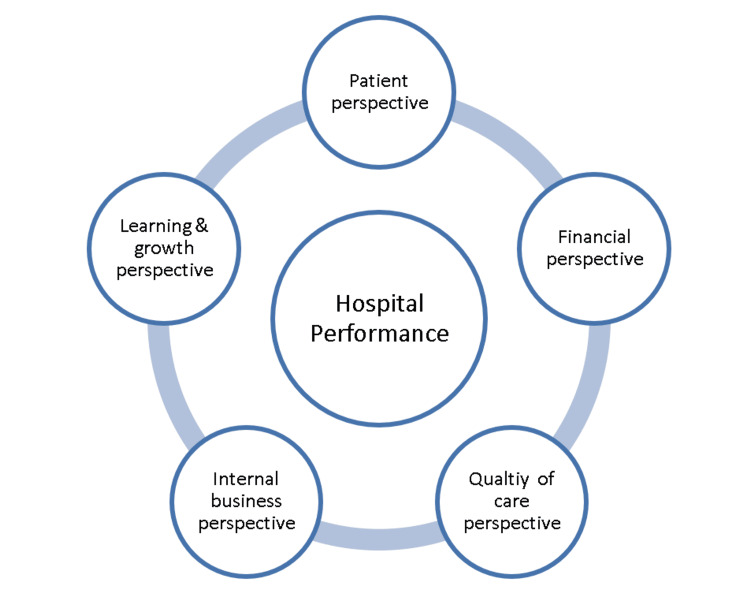
The proposed order of the BSC perspectives Image credits: Ahmad A. Abujaber

Similar to other business sectors, the adoption of the BSC concept in healthcare organizations has been significantly increasing [12, 13]. Consequently, Inamdar et al. [7] concluded that the BSC performance measurement and management system is appropriate for the healthcare organizations because (a) BSC helps the health organizations to develop market and customer-oriented strategies and to align the organization’s performance to the strategy; (b) it facilitates, monitors and assesses the implementation of strategy; (c) it helps assign accountability for performance at all levels of the organization, and (d) it facilitates continuous feedback on the strategy and enables the adjustments to the industry regulatory changes. Figure 1 illustrates the proposed order of the BSC perspectives in the strategic hierarchy.

Noticeably, all the frameworks designed to measure hospital performance share the same focus on the quality of care. For instance, Veillard et al. [14] emphasized measuring clinical effectiveness, which is concerned with delivering competent clinical care and achieving desired outcomes for all patients.

## Materials and methods

As a result, we attempt in this study to construct and validate a hospital performance measurement framework that adopts the BSC philosophy and considers the quality of care a separate dimension. Figure 1 shows the proposed BSC-based hospital framework.

The primary purpose of this study was to develop and empirically validate a comprehensive BSC-based framework to measure hospital performance. The construction of the BSC-based framework was grounded in a thorough literature review. The framework is multidimensional and consists of the four original perspectives of the BSC and the quality of care perspective that is particularly vital for healthcare institutions. This study was conducted in two phases: the first phase is an exploratory study that seeks feedback from healthcare practitioners and academicians to revise and identify the final framework and the performance measurement scale, which was empirically tested in the second stage. Secondly, the scale validity and reliability were conducted to determine the psychometric and theoretical validity of the performance framework as a construct in the hospital [9].

First phase: The exploratory study and development of the scale

After preparing the initial version of the framework, a group of expert academicians and practitioners were consulted to provide feedback about the constructed framework's face validity and its adequacy in measuring hospital performance. The experts' feedback was utilized to devise a performance scale in the second phase. As discussed by Elbanna et al. [9], an exploratory phase is needed when we know little about the subject under study. Therefore, the need for the exploratory phase in this study stems from the lack of an empirically valid hospital performance measurement framework that utilizes the BSC philosophy.

Second Phase: Scale's empirical validation

HMC has approved the project as a quality improvement project (MRC-01-18-286). The scale was presented in a questionnaire, and it was administered in English.

Sampling procedure: The initial sampling population consisted of 200 senior managers working at HMC in Qatar. Managers who had assumed any managerial role in HMC or other healthcare organizations and were aware of their hospitals' performance were targeted. The data was collected from the managers through an online survey using a standardized questionnaire with a 5-point Likert scale. The importance and benefit of this study were communicated to the participants by stating that in a cover letter. In addition, the participants were assured that confidentiality and anonymity would be strictly maintained.

## Results

The overall sample was 200 senior managers. Of the eligible sample, 81 participants responded by completing their questionnaires; thus, the response rate was 40%. The sample demographics of this study are illustrated in Table 1.

**Table 1:** Analysis of the general characteristics of the sample

Characteristics	Frequency	Percentage
Gender		
Male	46	57
Female	24	30
Prefer not to answer	11	13
Highest academic degree		
Bachelor's degree	21	26
Master's degree	56	69
PhD	4	5
Total years of experience in HMC (years)		
1 - 5	21	26
6 - 10	37	45
11 - 15	7	8
>15	9	11
Total experience in healthcare administration		
1 - 5	9	11
6 - 10	43	53
11 - 15	12	15
>15	17	21

The empirical validation of the framework includes the test of content validity, exploratory factor analysis, confirmatory factor analysis, convergent validity, discriminant validity, reliability tests, namely, Cronbach's alpha and composite reliability, and the correlation among the framework's dimensions [3,9].

Exploratory factor analysis (EFA)

Elbanna et al. [9] cited that "factor analysis is used to check whether indicators are gathered in the ways proposed by prior specifications of the specified dimensions". Therefore, exploratory factor analysis has been done to determine whether or not the scale items will load in the produced dimensions. As recommended by [15-16], the EFA was conducted using principal component analysis with varimax rotation and produced five factors.

Confirmatory factor analysis (CFA)

Confirmatory factor analysis (CFA) helps the researcher determine the multidimensional model's adequacy. A comparative analysis can be conducted between the four-factorial and the five-factorial models to identify the model suggested by the EFA (Table 2).

**Table 2:** Comparative analysis of models of various dimensionalities

Dimension model	x^2^	df
One	227.073	170
Four	131.339	116
Five	103.276	100

The proposed five-factor solution was introduced, including the innovation, customer, financial, learning, internal business, and quality perspectives (Table 3).

**Table 3:** Confirmatory factor analysis for BSC items

BSC items	Component				
	1	2	3	4	5
F1. Asset turnover	0.92				
F2. Return on investment	0.92				
F3. Operating income	0.71				
F4. Return on equity	0.61				
F5. Expense per service unit	0.57				
P4. Market share	0.55				
Q1. Preventable mortality rate		0.90			
Q2. Adverse events rate		0.78			
Q3. Hospital acquired infection		0.70			
Q4. Unplanned readmission within 48 hours		0.69			
IB1. In-hospital length of stay			0.91		
IB2. Timelines of care delivery			0.84		
IB3. Timeliness of patient discharge			0.81		
IB4. Bed utilization rate			0.73		
L1. Employee satisfaction				0.96	
L3. Employee training				0.94	
L4. Process improvement initiatives				0.61	
L5. Use of data warehousing				0.58	
P2. Complaints per 1000 patient					0.91
P3. Appreciation/complements per 1000 patient					0.87

Convergent validity

Convergent validity is the extent to which multiple indicators represent a common construct [3]. To establish the convergent validity of a specific construct, the factors loading needs to be statistically significant, i.e., 0.5 or greater [17]. Also, the construct has to have acceptable reliability, i.e., 0.70 or greater, and the average variance extracted (AVE) for any specific construct should be greater than 0.50 [3,9]. According to Hamann et al. [3], the AVE measure is the amount of variance in a set of indicators accounted for by the latent factor in the model. Table 4 shows that the minimum factor loading was more significant than 0.5, the minimum reliability was greater than 0.7, and the average variance extracted (AVE) was greater than 0.5 [18]. 

**Table 4:** Results of constructs’ reliability AVG: Average, *AVE: Average extraction

Constructs	Number of items	Cronbach’s alpha	Composite reliability	AVG loading	AVE* (AVG loading squared)
Financial perspective	6	0.89	0.87	0.71	0.51
Quality perspective	4	0.84	0.85	0.77	0.59
Internal business perspective	4	0.88	0.90	0.82	0.68
Learning perspective	4	0.88	0.86	0.77	0.59
Patient perspective	2	0.84	0.88	0.89	0.79

Discriminant validity

According to Sekaran [15], discriminant validity can be established when two or more distinct factors are not correlated. It is defined as the degree of divergence among indicators designed to measure different constructs. According to Elbanna et al. [9], to assess the discriminant validity, the square root of the AVE of each construct needs to be higher than the correlations between it and any other constructs in the model. Table 5 shows that the discriminant validity was established as the squared correlations among the five constructs were lower than any of the variances extracted (AVE).

**Table 5:** Discriminant validity results AVE = Average Variance Extracted

	Financial		Quality		Internal business		Learning		Patient	
Correlation	squared correlation	AVE	squared correlation	AVE	squared correlation	AVE	squared correlation	AVE	squared correlation	AVE
Financial			0.127	0.549	0.24	0.635	0.18	0.636	0.11	0.692
Quality					0.200	0.594	0.19	0.592	0.08	0.736
Internal business							0.283	0.550	0.05	0.691
Learning									0.167	0.650
Patient										

Reliability analysis

Reliability analysis is a prerequisite for validity analysis [3]; the reliability of a measure denotes the degree to which it is free of bias (error) and provides consistent measurement throughout time and across the many components in the instrument. This study utilized the Cronbach's alpha test to measure internal consistency and scale reliability, similar to [3,9]. However, due to the weakness of Cronbach's alpha in determining the true reliability as a sole measure [19], the Use of composite reliability can increase the robustness of the study's method and strengthen our determination of the scale reliability and validity. So, consistent with [9], the study conducted composite reliability and Cronbach's alpha tests. All variables were found to have alpha and composite reliability higher than 0.7.

Content validity

Content validity ensures that the measure includes an adequate and representative set of items that tap the concept [15-16]. According to Hamann et al. [3], content validity is established by providing a definition for the targeted construct and selecting theoretically and logically related items. The scale items were identified from the previous studies (Table 6). To ensure that the scale satisfies the content validity requirements, the judgment of the healthcare professionals has been incorporated in constructing the final version of the scale. Moreover, data collection from knowledgeable respondents enhanced content validity.

**Table 6:** Proposed Hospital Performance Measures and Indicators

Dimension	Measures/indicators	References
Financial perspective	Asset turnover, Return on investment, Operating income, Return on equity, Expense per service unit, Adherence to budget	[5, 9, 20-23]
Customer perspective (patient-centeredness)	Patient satisfaction, Complaints per 1000 patients, appreciation/complements per 1000 patients, market share	[10, 14, 21-25]
Quality of care	Avoidable mortality rate, Avoidable morbidity rate, Adverse events rate, Hospital-acquired infection, Unplanned readmission within 48 hours	[5, 14, 22, 25-27]
Internal business perspective (Operations effectiveness)	Length of stay, Timelines of treatment, Bed utilization, Time from door to therapy; Patient turnover	[5, 14, 21, 25, 28-31]
Learning and Growth (Employees and Innovation)	Employee satisfaction, Employees turnover rate, Employee training, Process improvement initiatives,i.e., implementation of electronic medical records, implementation of quality improvement principles (i.e., six sigma, lean), Use of data warehousing, business intelligence, and predictive analytics	[6, 9, 14, 22, 23, 32-42]

## Discussion

Performance measurement is a key interest in strategic management research. It is also crucial for achieving strategic goals and for an organization's competitiveness [6]. Despite the complexity and the different strategic orientation, hospitals need to have a robust system that enables the management to measure, improve, and drive performance [7]. The literature demonstrates that BSC is an appropriate philosophy to measure and improve the performance of hospitals and healthcare organizations. However, adaptation to satisfy the healthcare industry's uniqueness is still required. The original four perspectives are deemed appropriate for the hospital environment. By adding the quality of care perspective as a distinct dimension, as recommended by [5], BSC can achieve the required comprehensiveness [25]. We argue that the proposed BSC-based hospital framework is appropriate for hospital performance measurement. The framework satisfies the statistical validity and reliability requirements and, very importantly is, achieved the content validity requirement that healthcare managers and academicians assessed. Furthermore, this work supports the multidimensionality of the hospital performance, which is very important for the balanced approach that is essential in BSC philosophy.

This study provides evidence that BSC as a philosophy enjoys a high degree of adaptability to accommodate the specifics of every industry. In healthcare, quality of care is vital for the managers and care providers to understand how they perform and improve the care services and, ultimately, the patient treatment outcomes. 

It is essential to mention that the importance of performance measurement and improving efficiency in the healthcare industry is significantly growing. The advent of electronic health records, the advancement of data collection and storage technologies, and the modern computational capabilities, e.g., data warehousing, big data analytics, and machine learning, maximize the opportunities to utilize the massive data better than the healthcare industry generating. The recent publications showed that the utilization of the structured clinical data from the electronic health records has a tremendous positive impact on improving the functionality of the clinical departments [43]. This can be envisaged as an opportunity to enhance the timeliness of reporting performance, the objectivity, and the balance in the way we view, measure and improve the performance of the healthcare organizations.

However, the lack of high-quality publications that tackle the performance measurement in the hospitals presents a challenge for the researchers who aim to measure and improve hospital performance, particularly in the subject of the BSC. We relied on academic work in different sectors and industries to overcome this limitation. Hence, we faced other limitations, such as the different terminology and, very importantly, the various strategic orientations between healthcare and other business sectors. Healthcare organizations emphasize delivering clinical value while other businesses focus on economic values. Therefore, basing the hospitals' performance measurement system on the BSC philosophy helps achieve the desired balanced orientation.

Moreover, the hospital's level of care delivery (primary, secondary, or tertiary) presents another challenge because the strategic objectives differ following the hospital level and complexity. Therefore, no single framework can satisfy all hospitals' demands. Finally, the research that captures the differences between the profit and the nonprofit hospitals in terms of performance management is scarce. Therefore, this study aims to capitalize on the balance philosophy, adaptability, and customizability of the BSC to generate a framework that can be adapted to satisfy the needs of different hospitals with different orientations. Finally, Qatar is a small country with unique healthcare industry characteristics. Therefore, multicentric studies that evaluate healthcare industries in several countries, such as Gulf Corporation Council countries, would be of great value concerning the generalizability of the study findings.

## Conclusions

The five-dimensional BSC-based performance framework satisfied the statistical and logical requirements adopted to measure and improve hospital performance. The key contribution of this work is the development of a comprehensive and empirically validated performance measurement framework that enjoys a high degree of balanced orientation to enable the hospitals to attend to the needs and the expectations of the various stakeholders. Moreover, the focus on patient-centeredness coincides with the values of modern healthcare systems. Finally, the separation between the internal business perspective and the quality of care is necessary because the quality of care is viewed in this research as an outcome of multiple predictors, particularly the operations' effectiveness or the internal business perspective.
